# Gout management: Patent analytics and computational drug design explores URAT1 inhibitors landscape

**DOI:** 10.1371/journal.pone.0328559

**Published:** 2025-08-13

**Authors:** Jiaxin Zhang, Liang Hong, Wenfei Xu, Xin Zhang, Huina Fu, Xinan Song, Jing Zhao

**Affiliations:** 1 Joint Laboratory of Chinese Herbal Glycoengineering and Testing Technology, University of Macau & National Glycoengineering Research Center, Macao, China; 2 Macao Centre for Testing of Chinese Medicine, University of Macau, Macao, China; 3 State Key Laboratory of Quality Research in Chinese Medicine, Institute of Chinese Medical Sciences, University of Macau, Macao, China; 4 Department of Pharmaceutical sciences, Faculty of Health Sciences, University of Macau, Macao, China; Guru Nanak College, INDIA

## Abstract

Gout, caused by hyperuricemia, has a detrimental impact on patients’quality of life. The urate transporter 1 (URAT1) stands out as a key therapeutic target. However, its clinical development remains uncertain. This study aims to explore the landscape of URAT1 inhibitors by combining global patent analytics with computational drug design. We utilized the Derwent Innovation platform to analyze patents (from 2005 to 2024). Molecular docking was performed on 73.96% of novel compounds using AutoDock Vina. Additionally, scaffold diversity was analyzed using the Bemis-Murcko (BM) scaffold approach. A total of 2,195 entries were screened and eventually narrowed down to 1,056 high-value entries. The global research on URAT1 inhibitors is highly active, with China, the US, Japan, and Europe leading. Most patents are new compounds, indicating significant potential for novel drug development. Molecular docking showed ideal binding affinities for most compounds. The top five BM scaffolds were identified and compared with marketed drugs. This study highlights the potential for developing new URAT1 inhibitors. The identified compounds and scaffolds offer promising starting points for further drug development. Future work should focus on experimental validation and exploring clinical potential.

## 1. Introduction

Gout is the most common form of inflammatory arthritis [1]. Hypereuricemia (HUA) is a metabolic disorder related to purine metabolism in the body [2]. Elevated serum urate levels are associated with several metabolic conditions including increased body mass index, hypercholesterolemia, hypertriglyceridemia, elevated fasting glucose levels, and insulin resistance [3]. Prolonged high uric acid levels can lead to the deposition of monosodium urate crystals in joints or organs, which may overstimulate the body’s immune system and trigger gout, the most common form of inflammatory arthritis [4]. Purine nucleotides, nucleosides, and bases (such as adenine, inosine, and guanine) are metabolically degraded into uric acid, the end product of purine metabolism in humans due to the absence of functional uricase (urate-degrading enzyme) in the human genome [5,6]. Thus, serum urate levels depend on the balance between uric acid production and excretion, primarily regulated by renal mechanisms. The kidneys eliminate approximately two-thirds of urate daily [7], with the remainder excreted in feces.

The urate transporter 1 (URAT1), which is encoded by the SLC22A12 gene, belongs to the organic transporter (OAT) family and acts as an anion exchange uptake transporter located on the apical (brush border) membrane of renal proximal tubule cells. It mediates the reabsorption of uric acid from the proximal tubule, thereby playing a key role in uric acid homeostasis. URAT1 is predominantly expressed in the epithelial cells of the proximal tubules of the renal cortex [8,9]. It is believed that URAT1 can play an irreplaceable role in the reabsorption of urate by the kidneys [10]. More than 90% of patients with HUA have insufficient uric acid excretion [11], with URAT1 primarily responsible for this reabsorption [12]. Therefore, inhibition of URAT1 blocks the reabsorption of urate anions, thereby enhancing urate excretion [8,13]. Besides, a study [14] showed that URAT1 is a key regulator of the pathophysiology of metabolic syndrome and may become a new therapeutic target for individuals with insulin resistance, especially those with concomitant non-alcoholic fatty liver disease.

Urate-lowering drugs are mainly divided into two categories: [1] Xanthine oxidase inhibitors (XOIs). This type of urate-lowering drug can promote the reduction of uric acid synthesis rate. Typical drugs include allopurinol and febuxostat; [2] Drugs that increase uric acid excretion by the kidneys. Uricosuric agents inhibit renal transporters including URAT1 [15], thereby blocking the reabsorption of uric acid by the proximal convoluted tubule and promoting urate excretion. Although XOIs have shown good efficacy in the treatment of HUA and gout [16,17], they are accompanied by serious adverse effects, such as abnormal liver function and nausea [18]. In addition, a proportion of patients do not respond to XOIs, especially HUA caused by insufficient uric acid excretion, making it difficult to exert its therapeutic effect [19]. Therefore, considering that 90% of cases of HUA are due to reduced renal uric acid excretion [11], URAT1 may be a better treatment for gout relative to XOIs. URAT1 inhibitors can well complement the deficiencies of XOIs in xanthine clearance and the treatment of HUA [20]. Despite the recognized potential of URAT1 as a therapeutic target for HUA and gout [21], significant challenges remain in its clinical development [22], and there is a notable gap in the comprehensive analysis of the patent landscape and the potential drug value of URAT1 inhibitors [2,10,23]. Specifically, there is a lack of detailed research on the global distribution of patents, the key players in the field, and the structural diversity of the compounds being patented. This gap limits our understanding of the current state of URAT1 inhibitor development and hampers the identification of promising new compounds for further investigation [24].

This study aims to fill this gap by conducting a comprehensive analysis of the patent landscape of URAT1 inhibitors. Using the Derwent Innovation platform, we systematically analyzed 2,195 patents from 2005 to 2024, refining them to 1,056 high-value entries (inclusion rate of 48.11%). Our analysis focuses on temporal trends, countries/regions, key patent holders, and technical type. Additionally, we performed molecular docking, and analyzed scaffold diversity through the Bemis-Murcko (BM) scaffold. This series of operations aims to comprehensively present the innovative landscape of URAT1 inhibitors, outline clear research hotspots and development directions, and provide valuable reference guidance for subsequent scientific researchers to accurately select topics and conduct in-depth exploration in this field, helping to overcome the difficulties in the research and development of URAT1 inhibitors and promote their clinical applications to better benefit patients.

## 2. Methods

This study utilized the Derwent Innovation (https://www.derwentinnovation.com/login/) platform to search for patents. Derwent Innovation offers access to over 23 million essential inventions and more than 51 million patents from major patent offices, country-specific and proprietary sources, with global patent coverage and access to patent records from more than 50 patent issuing authorities. We searched for patents related to URAT1 inhibitors that were published prior to January 01, 2024. After searching and cleaning the data from Derwent, we used software and web pages such as Microsoft® Excel for Mac (version 16.77.1), Gephi (version 0.10.1) and Tubiaoxiu (https://www.tubiaoxiu.com/) to create the figures and tables.

### 2.1. Search strategies

In the search for patents related to URAT1 inhibitors, URAT1, its full name, synonyms, etc., are entered into the patent subject field of the database. The specific search strategy is as follows: CTB=(URAT1 OR (((uric acid) urate) NEAR2 (transporter OR exchanger OR (transport protein 1) OR SLC22A12 OR (solute carrier family 22 member 12) OR OAT4L OR organic anion transporter 4) NEAR50 (inhibit* OR regulat* OR modulat*)). The search yielded 1997 search records and 403 DWPI patent families. The search was conducted on January 11, 2024.

### 2.2. Criteria of inclusion and exclusion

The patent screening process involved a double-check by Zhang and Song. The same inclusion and exclusion criteria were applied during this process. The consistency of the double-check at the patent document level exceeded 80%, indicating a high level of precision and clarity in the inclusion criteria. The screening process is repeatable. After individual screenings, any disagreements regarding inclusion were discussed collectively. The patent screening criteria are as follows:

(1) Patents that only mention URAT1 inhibitors and are not the main drug introduced (e.g., drug combinations using either a URAT1 inhibitor or an XOI).
(2) Drugs, drug combinations, etc., that can only be used as food or health care products (unless the abstract or other specific descriptions indicate they can be used as drugs or pharmaceuticals).
(3) Patent with incomplete content.
(4) Preparation processes, manufacturing methods, improvement method (chemical industry), etc., of URAT1 inhibitors (new).
(5) Patents for URAT1 inhibitor (*in vitro*) screening and model construction.
(6) Patents related to the URAT1 gene without inhibitory effects.
(7) Patents related to the regulation of uric acid levels but not related to URAT1 inhibitors.
(8) Patents for non-human research.
(9) Obviously irrelevant patents.

The following descriptive analysis is based on the patent information of the family, including basic (published) patent number, patent publication year, patent publication country, patentee, title, abstract, International Patent Classification, application details, and citation information of all family members.

### 2.3. New compounds analysis

The compounds used for molecular docking in this study were derived from patents categorized under the “new compound” technology type. Since the compound structures typically protected in patents are often core structures with numerous substituents, we selected representative structures for molecular docking. When choosing substituent groups, the following principles were applied: 1. If a clear compound structure is present in the patent, the structure from the patent will be used. If there are multiple structures, the first one will be preferred. 2. If there are clearly preferred groups in the patent, select the preferred groups, giving priority to those ranked first. 3. Prioritize groups with simpler structures (e.g., among -CH3, -NH2, -OH, give priority to -OH). 4. Prioritize atoms with higher-ranking elements (e.g., between O and S, give priority to O). 5. The substituent position should preferably be in the ortho position. 6. If the number of rings is not specified, a six-membered ring will be used.

Molecular docking technique was employed to investigate the interactions between various molecules and the active sites of specific proteins, aiming to identify the essential structural features that influence their binding efficiency [25–27]. The three-dimensional structure of URAT1 (UniProt id: Q96S37) was predicted using the Alphafold program available on uniprot (https://www.uniprot.org/). The validity of the protein structure was verified using SAVES v6.0 (https://saves.mbi.ucla.edu/) and Ramachandran plots [28]. The software used for docking was AutoDock Vina, a widely utilized molecular docking program that combines a global search algorithm with local search optimization. Its scoring function employs a hybrid empirical force field to estimate binding affinity in units of kcal/mol, where a lower score indicates a stronger predicted binding interaction [29]. Before docking, the target protein needed to be dehydrated, polar hydrogen added, and saved as a pdbqt format file. The docking box was designed to encompass the potential bonding pocket as much as possible. The parameter values in the configuration file were set as exhaustiveness = 8, energy_range = 3, num_modes = 9. AutoDock Tools (ADT) from the MGL software package was used to convert pdb into pdbqt format for inputting proteins and ligands [30]. After the completion docking, both the macromolecule and ligand structures were saved in pdbqt format needed by Accelrys Discovery Studio (version 4.1) to explore and visualize the docking results and search for nonbonding interactions between ligands and amino acid residues of receptor protein [31].

A more detailed analysis and classification of the compounds were also conducted to uncover the characteristics of their scaffolds. BM scaffold analysis was performed on the compounds interacting with the core targets. First, the Extended-Connectivity Fingerprints (ECFPs) algorithm was applied to extract the ECFPs of the targeted compounds [32]. Similarity was assessed based on the Morgan fingerprint of the molecule, yielding the Tanimoto index of the two molecules. Subsequently, similar compounds were clustered using Taylor-Butina clustering, using a Tanimoto distance threshold of 0.5 [33,34]. Finally, the clusters were manually adjusted according to the similarity of the scaffolds.

## 3. Results and discussion

### 3.1. Results after searching and cleaning

**Fig 1:**
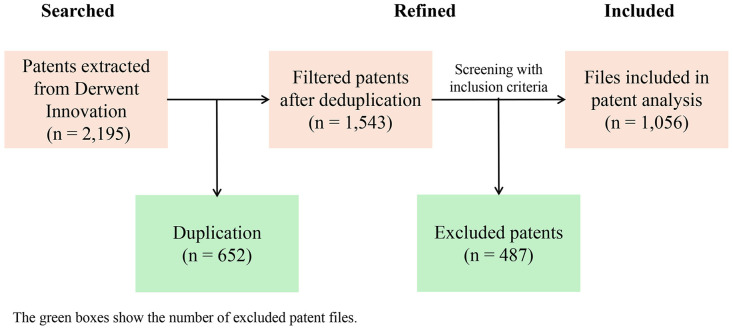
Patent screening process flow chart.

The search results yielded 2195 search records and 449 DWPI patent families. After further data cleaning, we obtained 1,056 included patents, 487 excluded patents, and identified 652 duplicate patents, resulting in an inclusion rate of 48.11%. The patent screening process is shown in the Fig 1. For a full list of search patent results, please see the S1 File.

### 3.2. Time trend

**Fig 2:**
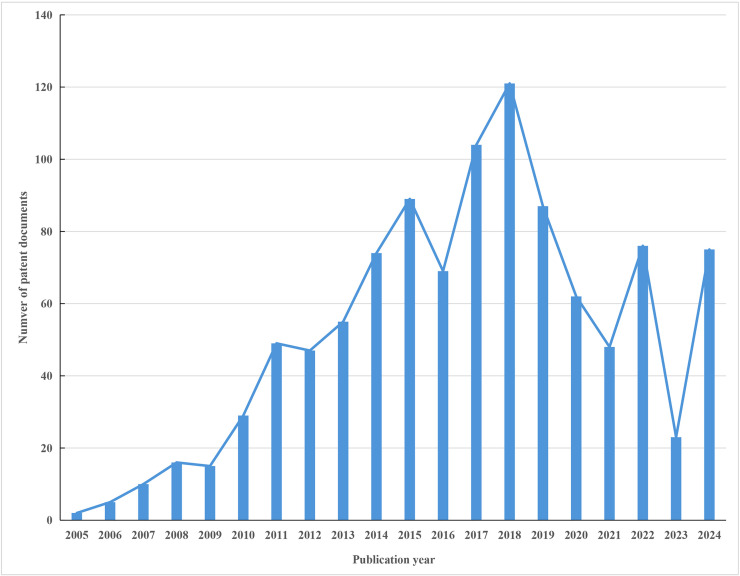
Annual distribution of patent disclosures related to global URAT1 inhibitors.

As shown in Fig 2, global patents related to URAT1 inhibitors first appeared in 2005. From 2005 to 2009, the number of patents remained consistently low, generally below 20. Between 2009 and 2018, there was a period of rapid growth in global URAT1 inhibitor-related patents. Despite occasional declines, the overall trend was upward, peaking in 2018 with nearly 120 patent disclosures annually. After 2018, the number of patents decreased, but there was a slight rebound in 2022. Countries around the world continue to closely monitor and research content related to URAT1 inhibitors.

### 3.3. Countries and regions analysis (distribution and changes)

**Fig 3:**
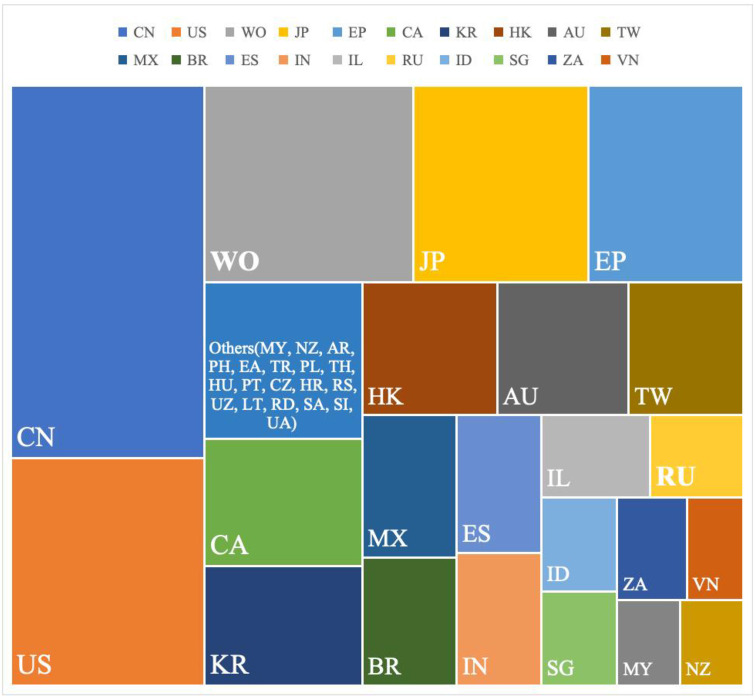
Comparison of the number of published patents in each country/regions.

This study encompasses patents published across 39 countries. Fig 3 illustrates a comparison of the top twenty countries/regions based on the number of published patents. The combined total of published patents from these top twenty countries/regions constitutes over 90% of all patents, ensuring the analysis holds universal representative significance and effectively reflects the global publication landscape of URAT1 inhibitor patents.

Table 1 displays the top ten countries/regions with the most published patents related to URAT1 inhibitors since 2005. It is evident that the majority of worldwide published patents are concentrated within these top ten countries/regions. Among them, the top three countries/regions (China, the United States, and the World Intellectual Property Organization) account for over 50% of the published patents, establishing their dominant position. URAT1 inhibitors are experiencing rapid development in these leading countries. Consequently, in the subsequent patent family analysis, priority is given to utilizing World patents to represent patents within a specific patent family, followed by U.S. and Chinese patents.

**Table 1:** Ranking of global URAT1 inhibitor-related patent disclosure countries/regions.

Rank	Country/region	Numbers	Proportion
1	China (CN)	193	18.28%
2	United States (US)	105	9.94%
3	World Intellectual Property Organization (WO)	92	8.71%
4	Japan (JP)	84	7.95%
5	European Patent Office (EP)	74	7.01%
6	Canada (CA)	47	4.45%
7	Korea (KR)	45	4.26%
8	Australia (AU)	45	4.26%
9	Hong Kong (HK)	42	3.98%
10	Taiwan (TW)	34	3.22%

**Fig 4:**
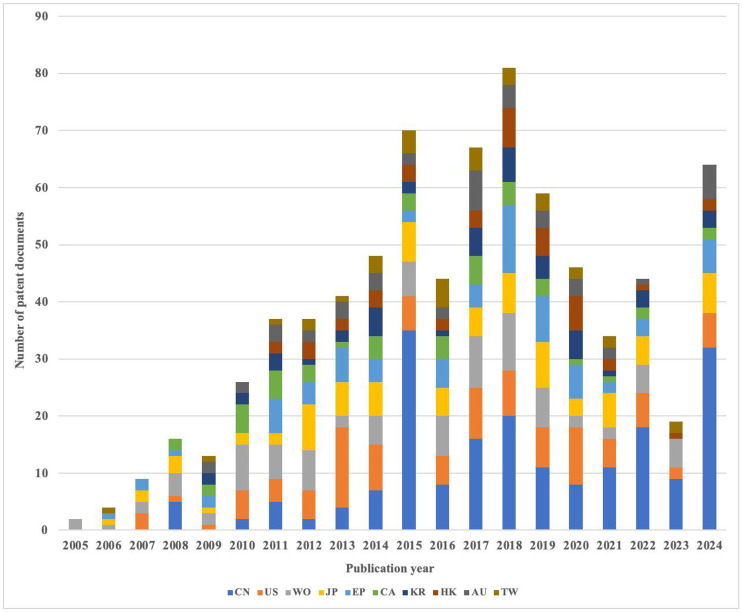
Changes in the number of patents from the top ten published countries/regions over the years.

The annual changes in patent disclosure volume for the top ten countries/regions are presented in Fig 4. In terms of quantity, the United States and China are two countries that have a significant influence on URAT1 inhibitor patents. The United States was the first to begin research on URAT1 inhibitors before China. Before 2015, the number of URAT1 inhibitor patents in the United States was much higher than that of China. However, China, as a rising power, started to increase its research on URAT1 inhibitors after 2015, and the number of patents published in subsequent years revealed an absolute advantage worldwide. In addition, Japan and Europe also place great importance on URAT1 inhibitor research.

**Fig 5:**
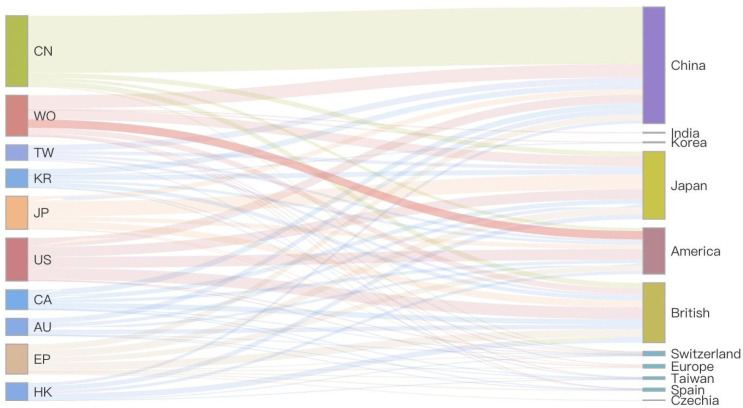
Sources of patent holders in the top ten countries/regions with the number of published atents.

To investigate the relationship between patentee countries and patent disclosure countries, Fig 5 illustrates the sources of patentees for patents originating from the top ten countries with the highest number of published patents. The primary sources of patent holders globally are China, Japan and the United States. A majority of the patents disclosed in China belong to Chinese patentees, with Japan following behind. In contrast, the United States and the United Kingdom tend to disclose patents in various countries/regions. The proportion of British patent holders in the United States is similar to that of American patent holders. However, not many American patent holders choose to publish their patents in the United States. For most patent applicants, due to the challenges of international application, their preferred target country for patent application is their home country. Alternatively, they may have their patent enter another country by first applying for a world patent.

### 3.4. Analysis of the assignee and URAT1 drugs

In addition to the country of the assignees, we also delved into their detailed information. Table 2 presents the top ten patent owners based on the number of patent families, the number of patents they hold, and the status and IC50 of their compounds. Among them, AstraZeneca, which ranks first, possesses significantly more patents than other patentees. Furthermore, among the top ten patentees, the country/region with the most affiliations is China (4 assignees), followed by Japan (3 assignees), the United States (2 assignees) and the United Kingdom (1 assignee). Although the UK has a relatively small number of patent holders, it boasts an absolute advantage in the number of patents it holds (216 patents). In terms of patent families count, ZHANG YUAN-QIANG from China has the largest number of patent families, but each patent family contains only one patent on average, making him the patentee with the lowest average number of patent families per patent family. When examining the types of patent holders, it is evident that company-developed patents hold a dominant position over university-developed ones.

**Table 2:** The top ten assignees in the world in terms of number of patents related to URAT1 inhibitors.

Rank	Assignee	Patent documents	Patent families	Country/ region	Type	Compound from the assignee	Status of the compound	IC50 of the compound (μM)
1	ASTRAZENECA PLC	216	16	Britain	Company	Lesinurad	discontinued	47.76
2	TEIJIN LTD	83	12	Japan	Company	/	/	/
3	KISSEI PHARMACEUTICAL CO LTD	70	8	Japan	Company	/	/	/
4	WELLSTAT GROUP OF COM-PANIES	54	5	America	Company	/	/	/
5	JIANGSU HENGRUI MEDICINE CO LTD	36	10	China	Company	/	/	/
6	ZHANG YUAN-QIANG	34	34	China	Individual	/	/	/
7	JIANGSU ATOM BIOSCIENCE & PHA-RM CO LTD	34	14	China	Company	/	/	/
8	ARTHROSI THERAPEUTI-CS INC	24	2	America	Company	AR882	Phase Ⅲ	1.18
9	SHANGHAI YINGLI PHAR-M CO LTD	24	5	China	Company	YL-90148	Phase Ⅲ	None
10	SHANGHAI FOCHON PHA-RM CO LTD	22	4	Japan	Company	/	/	/

We carried out an in-depth background investigation on the top ten patent holders. The first is AstraZeneca Pharmaceutical Company, which ranks first. Their research on URAT1 inhibitors mainly centers on lesinurad. In December 2015, lesinurad was approved in the United States as a combination therapy with a XOI. Lesinurad is indicated in adults for use in combination with an XOI (such as allopurinol or febuxostat) to treat patients with gout (with or without tophi) who have high uric acid levels that are not adequately treated with XOIs alone. Therefore, AstraZeneca has an absolute advantage in URAT1 inhibitors. Next is ARTHROSI THERAPEUTICS INC, a clinical-stage biotechnology company focused on developing treatments for gout and chronic kidney disease. They pioneered the clinical development of a first-generation urate inhibitor treatment for gout at Ardea Biosciences, which was ultimately acquired by AstraZeneca for $1.26 billion. A powerful uricosuric drug called AR882 is being developed. A phase 2b randomized study to evaluate the safety and efficacy of AR882 versus placebo in gout patients has been completed. AR882 is being evaluated versus allopurinol for the treatment of tophi gout. Phase 2 randomized study of safety and efficacy in patients. Finally, there is Shanghai Yingli Pharmaceutical Co., Ltd, which has a URAT1 inhibitor with a research and development number YL-90148, which is currently in the second phase of clinical trials. Except for these three patent holders, none of the others are developing URAT1 inhibitor drugs. In addition, in the research of HUA, TEIJIN LTD and Jiangsu Hengrui Pharmaceutical Co., Ltd. have not developed URAT1 inhibitor drugs, but they have FEBURIC febuxostat, a XOI drug.

When conducting background checks on all patent holders, we found that there is a company called Urica Therapeutics in addition to these ten patent holders, which focuses on the development of the URAT1 inhibitor drug Dotinurad. In May 2021, Urica reached an agreement with Fuji Yakuhin Co. Ltd. to develop Dotinurad in North America and Europe. Dotinurad (URECE® tablets) was approved in Japan in 2020 as a once-daily oral therapy for the treatment of gout and HUA. In a Phase 3 clinical trial, dotinurad was effective and well-tolerated in more than 500 Japanese patients treated for up to 58 weeks. More than 1,000 Japanese patients have been safely treated with Dotinurad, and the drug is currently undergoing Phase 3 clinical trials in China.

Tracing the history of URAT1 inhibitors, Before the identification of URAT1 in 2002, probenecid, sulfinpyrazone, and benzbromarone had long been used for the treatment of hyperuricemia; it was then recognized that their uricosuric effect stemmed from the inhibition of URAT1 activity [35]. However, there were serious problems in the clinical administration of these drugs because most of them displayed low efficacy and serious side effects. As a result, these drugs are constantly being phased out and replaced by new inhibitors. At present, in addition to Lesinurad developed by ASTRAZENECA PLC and Dotinurad developed by Fuji Yakuhin mentioned above, the existing URAT1 target-positive drugs include Arhalofenate and Verinurad. Among them, Arhalofenate has been shown to inhibit uric acid transport as well as preventing inflammation and pyroptosis via activating PPARγ thereby blocking caspase-1 activation of HUA *in vitro* [36]. Verinurad may cause adverse renal reactions when used alone [37], so its combined use with other uric acid-lowering drugs is currently being studied more (for example, patent WO2021009197A1 introduces a pharmaceutical composition containing verinurad).

### 3.5. Analysis of the technical type

**Fig 6:**
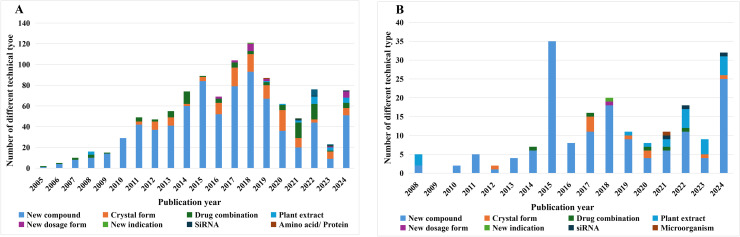
Changes in technology types by year in the world (A) and in China (B).

Based on the patent content, we divided the 1056 patents retrieved and finally included into 10 different technology types, and produced a chart of changes in different technology types over the years as shown in Fig 6A. Regardless of the proportion of the number or the year-on-year changes in the numbers, the technology type of URAT1-related patents has been dominated by new compounds. After 2008, the technology types of patents began to become more and more complex, but new compounds still accounted for more than half. The second largest number of patents are crystal morphology patents, but they appeared relatively late. It was not until 2011 that the first patent related to crystal morphology was published.

We conducted a specific analysis of the technology types of patents in China, the country with the most URAT1 inhibition patents. China’s patent types are the most diverse, including 7 types of technologies: new compounds, drug combinations, plant extracts, crystal forms, siRNA, new indications, and new dosage forms. A technology type chart of Chinese patents was made to more intuitively observe the distribution and changes (trends) of Chinese patents, see Fig 6B. It can be seen that after 2015, the types of patents began to diversify, and many patent types (such as drug combinations, crystal forms, etc.) are based on new compounds. Therefore, we can infer that as long as new compounds are continuously produced, there will be subsequent technological changes based on different new compounds. 161 patents have been disclosed, of which 126 are new compounds, followed by drug combinations [17] and plant extracts [10]. China was the first country to start researching plant extracts in 2008 and has been researching them up to the patent search date. This is inseparable from China’s long-standing research background in traditional Chinese medicine. China has a large population base, and the weighted prevalence rates of HUA and gout in Chinese adults were 17.7% and 3.2%, and is increasing year by year [38]. Take Benzbromarone, a URAT1 inhibitor, for example. It is a uricosuric agent, serving as a first-line urate-lowering drug according to the Chinese gout management guideline and a Class B drug in China’s National Medical Insurance Drug Catalog. It is also the most prescribed urate-lowering drug in China [39]. It is still a recommended drug in the “Guidelines for the Diagnosis and Treatment of Gout in China” and one of the main anti-gout drugs sold in the Chinese market. Therefore, URAT1 inhibitors have great development potential in China.

### 3.6. Analysis of new compounds

Our analysis encompassed dual inhibitors targeting xanthine oxidase (XOD) and URAT1, molecular docking and analysis of BM scaffolds.

#### 3.6.1. Dual inhibitors targeting XOD and URAT1.

Upon careful examination of the patented new compounds, we identified certain patent families that reported dual inhibition of XOD and URAT1. These patents are as follows: WO2018009615A1, WO2015123003A1, WO2008126899A1, WO2008126898A1, WO2010044404A1, WO2010044411A1, WO2010044410A1, CN114805192A, CN113087683A, CN110204494A (the above patent numbers are representative patents of the patent family). Due to the advantages of dual inhibition, research and development in this area have always been a priority. Examples include salinomycin [40], digallic acid [41], and a new compound named compound 27 [42]. Therefore, patents related to dual inhibitors should be the focus of attention.

#### 3.6.2. Molecular docking.

*In silico* analysis is a fast and inexpensive way to screen and obtain compounds with the biological activity required for further research [43,44]. Molecular docking, a type of computer analysis, is now widely used in the drug discovery process [45]. And the results of molecular docking are often directly analyzed [46–49]. In the process of using molecular docking for virtual screening, the docking software usually gives the binding free energy for each docking result, reflecting the possibility of ligand-receptor binding. Lower binding free energies correspond to higher affinities and stronger potential binding interactions, which indicates the greater the possibility of becoming a potential drug [50].

**Fig 7:**
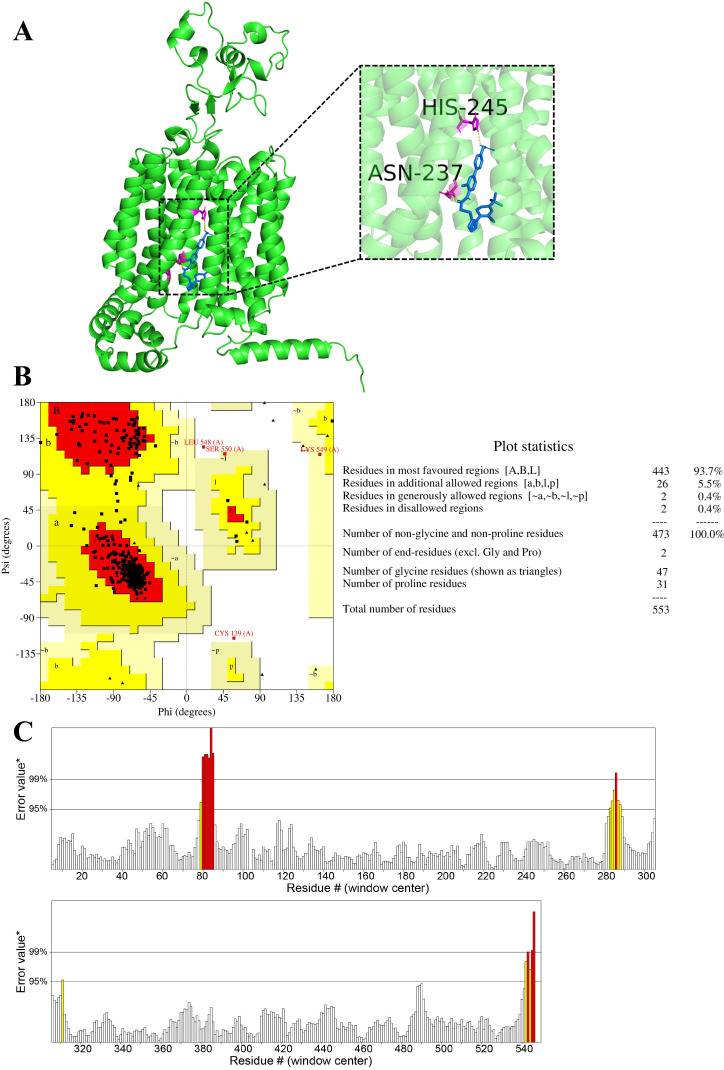
The connection mode between compound 1 in Table 3 and URAT1 protein.

In this study, the three-dimensional structure of URAT1 was obtained using the AlphaFold program in UniProt, as shown in the protein section in Fig 7A. Ramachandran plots were generated to analyze the distribution of amino acid residues in the URAT1’s 3D structure. Generally, a protein structure is deemed acceptable if over 90% of its amino acid residues lie within allowed regions [51]. The Ramachandran plot results (99.6% of amino acids in allowed regions) confirmed the protein’s conformational integrity and the model’s reliability, as shown in Fig 7B. The ERRAT method was also used to assess the overall quality of URAT1’s crystal structure, yielding a score of 96.475%, as shown in Fig 7C. These results collectively validate the reliability of the URAT1 model as a protein receptor.

**Table 3:** Patent and compound structures with docking thresholds less than −9.

Rank	Patent number	Compound type	Docking thresholds (kcal/mol)
1	US8372807B2	Substituted N-phenyl-acetamide compounds	−10.1
2	CN106831556B	Sulfonamides	−9.7
3	CN104262276B	Compounds containing benzene tetrazole acetic acid	−9.6
4	WO2010044410A1	Biarylisonicotinic acid derivatives	−9.6
5	CN116570584A	salinomycin	−9.4
6	TWI666201B	Fused ring derivatives	−9.3
7	CN104292175B	2-Methyl-2H-tetrazole derivatives	−9.3
8	CN106008488B	Cyanoindole derivatives	−9.2
9	WO2010044405A1	Fused ring derivatives	−9.2
10	CN104292177B	1,5-diaminonaphthalene derivatives	−9.2
11	CN104311452B	nitrile naphthyl cyclosuccinic acid amide	−9.2
12	CN104292123B	Phenylnaphthylenesuccinamide derivatives	−9.2
13	CN104327000B	Phenyl-substituted triazolesulfinylmalonic acid compounds	−9
14	CN104370842B	Sulfonylmalonic acid compound substituted phenyltriazole	−9
15	CN104370843B	Halogenated sulfenylmalonic acid compounds	−9
16	CN104311441B	Chlorinated naphthalene cyclosuccinic acid amide derivatives	−9

*Note*. The chemical structures are shown in Fig 8.

**Fig 8:**
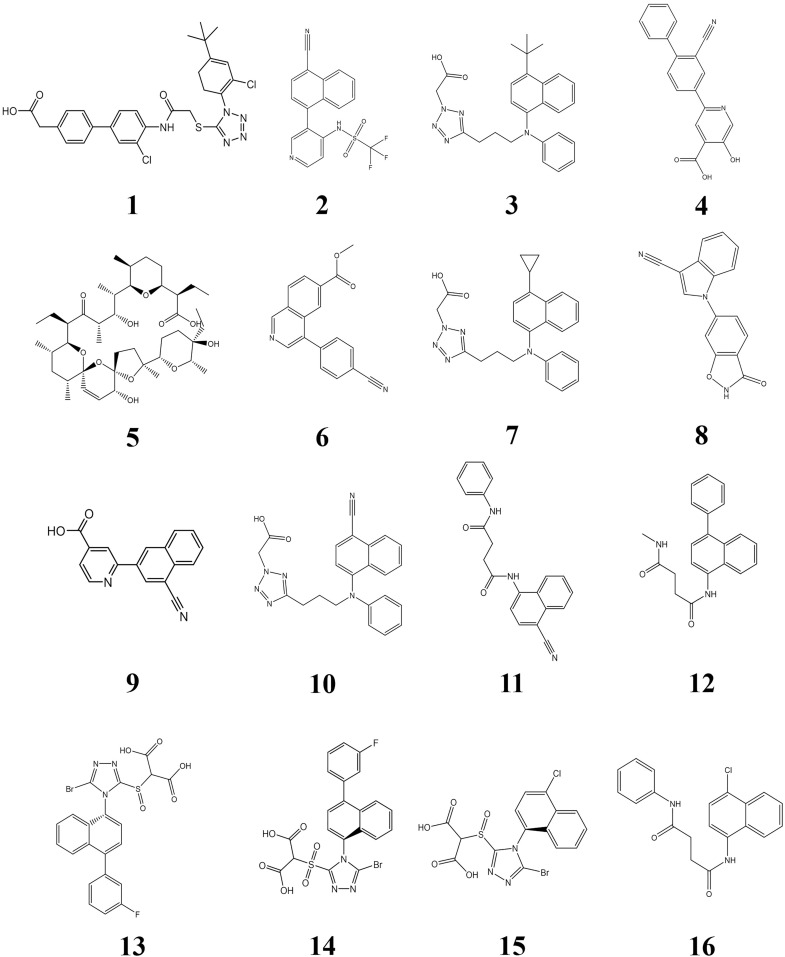
Chemical structures of 16 compounds with binding free energies of less than −9 kcal/mol to URAT1 protein.

Due to the technical type characteristics of URAT1 inhibitor patents (mainly new compounds), this article collected 727 structures involved in new compound patents. Molecular docking was conducted to verify the affinity of different compounds for the URAT1 protein receptor. Among them, 16 compounds have binding free energies of less than −9 kcal/mol. As shown in Table 3, these 16 compounds have high drug potential, and their structures are shown in Fig 8. The patents they belong to, as well as other compounds with the same core but different substituents included in the patents, also warrant particular attention. Fig 7A uses the first compound in Table 3 as an example to show how the compound is connected to URAT1. Compound 1 forms a hydrogen bond with the His245 and Asn237 amino acids of URAT1. Hydrogen bonding between polar groups within tightly packed folded proteins is more favorable than similar interactions with water in unfolded proteins, and polar group burial contributes significantly to protein stability [52]. The presence of hydrogen bonding therefore stabilizes the association between the compound and URAT1.

#### 3.6.3. Analysis of BM scaffolds.

**Fig 9:**
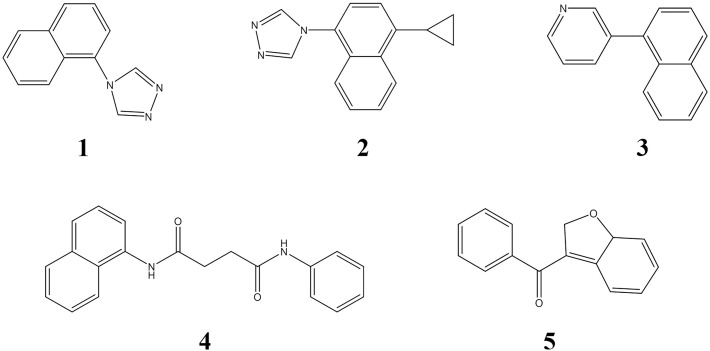
The chemical structures of the top five BM scaffolds.

In addition, we simplified the compounds to their common BM scaffolds to quantify the scaffold diversity of patent compounds. These scaffolds were classified into 77 categories according to a threshold of 0.5 [53] and followed by manual adjustment. The top five scaffolds were then compared with the structures of marketed (or once marketed) drugs (Lesinurad, Verinurad, Benzbromarone and Dotinurad). The chemical structures of these five BM scaffolds are shown in Fig 9. Table 4 shows the same or similar comparison results.

**Table 4:** Comparison of the structure of patented compound scaffold and marketed drug.

No.	Numbers	proportion	Comparison with marketed drugs
1	15	10.27%	–
2	9	6.16%	Lesinurad
3	9	6.16%	Verinurad
4	4	2.74%	–
5	4	2.74%	Benzbromarone/Dotinurad

*Note*. The chemical structures of the BM scaffolds are shown in Fig 9.

All these scaffolds all contain more than two rings, and most of them contain heteroatoms, so their molecular weight is about a few hundred Daltons. Further analysis shows these skeletal structures are closely related to key pharmaceutical properties like drug activity and metabolic characteristics. For example, multiple rings may offer broader target-binding sites, and heteroatoms can raise molecular polarity, improving drug solubility and thus promoting drug absorption and distribution [54].

There are also other marketed drugs (such as Arhalofenate) whose BM scaffolds have appeared in patents, and its patent number is CN108727238A, but because this scaffold only appears once, it is not listed in Table 4. These compounds with the same scaffold covered by these patents are worth further exploration. From a drug development viewpoint, this exploration can expand the chemical space for drug research, raise the likelihood of discovering new effective drugs, and offer convenience for follow-up R & D by providing similarities and references in synthesis and quality control.

It is worth noting that although there is no drug on the market for the first scaffold, according to the information shown in the patent, it can be seen that the first scaffold is a modified version of the Lesinurad scaffold, replacing the original three-membered ring with a nitro substitution, which may further improve the efficacy or adverse reactions of Lesinurad. It’s a significant direction for drug development. Rational modifications to existing drug scaffolds can yield better – performing new drugs, offering stronger tools for clinical treatment and improving patients’ outcomes and quality of life.

The fifth scaffold has two drugs that have been on the market, and they were developed by different companies (Benzbromarone is Sanofi-Synthélabo, Dotinurad is Fuji Yakuhin). This suggests the scaffold’s great value. In drug development and market competition, it indicates these drugs have a broad application prospect and demand. The R & D investment and market promotion by different companies can drive continuous innovation and optimization of drugs in this field, offering patients more options. It also encourages R & D institutions to explore new directions and strategies for further development of scaffold – based drugs, enhancing product competitiveness and market share.

### 3.7. Citation relationships between patent families

**Fig 10:**
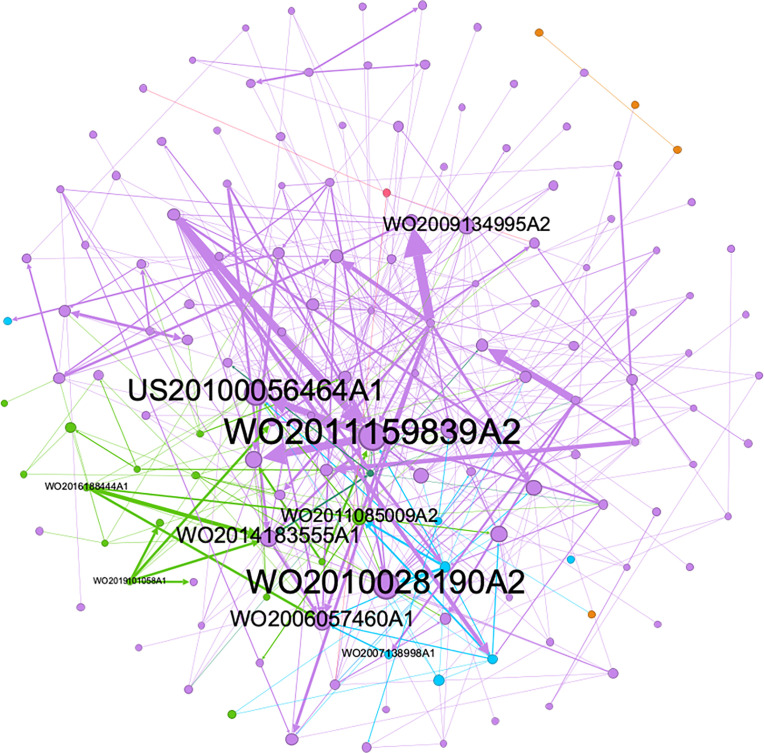
Citation relationship diagram between patent families.

Adopting a network perspective, the research focus shifts from individual patents to citation relationships among patent families. The network is constructed by Gephi to observe the internal reference relationships between them. Each node represents a patent family, the directed edge corresponds to the citation relationship, and the arrow represents the citation direction. Nodes are spatially distributed using the Fruchterman-Reingold method, a force-directed layout algorithm. The node size is set according to its in-degree value, i.e., the larger the in-degree value, the larger the node size and the more times a given patent is cited. Paint the nodes different colors based on different technology types. Purple represents new compounds, light green represents crystal forms, blue represents drug combinations, orange represents plant extracts, dark green represents new dosage forms, and pink represents siRNA. Fig 10 shows the representative publication numbers of patent families that are cited frequently and will be mentioned later. In the entire network diagram, patent families of new compound types (purple) are cited most frequently, accounting for the vast majority, followed by crystal forms (light green) and drug combinations (blue), which is related to the fact that these three technology types have the largest total number. Among them, the parent nucleus of WO2011159839A3 (which publicated by ASTRAZENECA PLC) is consistent with lesinurad invented by it. Therefore, there is reason to believe that its high frequency of citations comes from the development and clinical use of lesinurad.

In terms of reference relationships, it is not difficult to find that the references between the same technology types are much larger than the references between different technology types. But among the references between these different technology types, we can find some patterns. Taking the reference relationship between WO2014183555A1 (new compound) and WO2016188444A1 (crystal form) as an example, some researchers selected one of the sodium salts (1-((6-bromo-quinoline-4-yl)thio)cyclobutyl) from the compounds disclosed in the former) and made I-type crystal. The I-type crystal of the compound obtained in the latter has a good crystal form stability and chemical stability, and the crystallization solvent used has a low toxicity and low residue, and can be better used in clinical treatment. There is also WO2019101058A1, which also modified the crystal form of certain compounds based on the compounds disclosed in WO2014183555A1, and obtained compounds more suitable for clinical use. In addition, the citation relationship between WO2006057460A1 (new compound) and WO2007138998A1 (drug combination) is also worthy of attention. WO2007138998A1 introduces a pharmaceutical composition consisting of the compound in WO2006057460A1 and one or more pharmaceutically acceptable additives. They have more potent inhibitory action on URAT1 activity as compared to conventional URAT1 activity inhibitors and have no or very weak CYP inhibitory action, wherein the time-course stability of the active ingredient during manufacturing process or storage thereof has been improved, or show rapid dissolution property (disintegratability), or simultaneously have these characteristics. The citation relationships between patents of different technology types show us the peak period of technology update from 2006 to 2022. Because of technology updates, there will be mutual citations of patents between different technology types. This is consistent with what was mentioned above. The period of rapid development of URAT1 inhibitor patents from 2009 to 2014 coincides with the period.

## 4. Limitations and regulatory challenges

### 4.1. Limitations

There are some limitations in our research on URAT1 inhibitors. Due to the rapid increase in the number of patent applications, we did not cover all relevant patents around the world. Also, due to technical limitations, information about some patentees and their invented drugs is not fully disclosed, so the information about patentees may not be comprehensive or complete. Our analysis of URAT1 inhibitors through patents has inherent limitations. Specifically, not all active URAT1 inhibitors may be captured in patent databases, particularly those that are still in the early stages of development or have not yet been patented. Moreover, molecular docking is vital for initial compound screening, yet its predictions carry uncertainty and limitations. Experimental validation is essential to accurately evaluate their biological activity and pharmaceutical value.

### 4.2. Regulatory challenges

The regulatory challenges for URAT1 inhibitors mainly lie in the following aspects: Regional differences: There are significant differences in drug approval standards and procedures across countries and regions. For example, benzbromarone, which is widely used in some Asian countries, is banned in the United States primarily due to its hepatotoxic potential [55]. In Europe and Japan, some URAT1 inhibitors have been approved and marketed, but the approval process in the United States is more stringent, requiring more clinical data to demonstrate their safety and efficacy. Clinical trial requirements: To gain market approval, URAT1 inhibitors need to undergo multi – stage clinical trials to assess their safety and efficacy in different patient populations. These trials are not only time – consuming and costly but also need to meet strict ethical and scientific standards. For instance, Verinurad, currently in Phase III clinical trials, has shown high selectivity and efficacy but still needs to complete all necessary clinical trial steps to obtain approval [56]. Safety and side effects: Early URAT1 inhibitors such as Benzbromarone and Lesinurad have some safety and side – effect issues that limit their clinical application [57]. Although new – generation URAT1 inhibitors have made significant improvements in selectivity and safety, regulatory authorities still need to carefully evaluate the potential long – term risks of their use. Drug interactions: URAT1 inhibitors may interact with other drugs, especially in the context of polypharmacy. For example, Lesinurad needs to be used in combination with XOIs (such as Allopurinol) to enhance its efficacy and safety [58]. This combination therapy requires additional clinical research to verify its applicability and effectiveness in different patient populations. Structural and mechanistic studies: Despite important progress in recent years in the structural and mechanistic studies of URAT1, the lack of complete structural information remains a major obstacle to the development of highly selective inhibitors. For example, the instability of URAT1 makes it difficult to resolve the structure of the wild type, which limits the in – depth understanding of the drug’s mechanism of action [13].

## 5. Conclusion

This study provides an overview of the development of URAT1 inhibitors by analyzing the changes in patents related to URAT1 inhibitors over time, as well as the analysis of patents by countries/regions, key patentees, and patent family representatives. Research on URAT1 inhibitors in various countries/regions around the world, led by China, the United States, Japan, and Europe, is still at a high level. From a patent perspective, URAT1 inhibitors, as an emerging drug that started late and developed rapidly, still have large room for development in the future, which is inseparable from its steadily growing clinical demand. Especially when XOI, a commonly used drug for HUA, is often accompanied by serious adverse reactions, more and more researchers are beginning to pay attention to uricosuric monotherapy or combining an XOI with a uricosuric, which is also a uric acid-lowering treatment recommended by the American College of Rheumatology Guideline for the Management of Gout. The patent landscape for URAT1 inhibitors is overwhelmingly dominated by novel compounds, which underscores the vast, yet-to-be-explored potential for the creation of innovative URAT1 inhibitor drugs. When juxtaposed with the URAT1 inhibitors that are currently available on the market or undergoing clinical trials, the compounds uncovered in this study exhibit highly promising characteristics. Historically, many approved URAT1 inhibitor drugs have encountered significant challenges, often being withdrawn from the market or discontinued due to severe adverse effects (for instance, Probenecid and Benzbromarone are known to cause severe hepatotoxicity and gastrointestinal intolerance). As a result, safety has emerged as the paramount concern in the development of these drugs. Pharmaceutical research and development enterprises have the opportunity to modify the original drug structure based on the analysis and research of BM scaffolds. They can also consider combining these drugs with XOIs to mitigate the toxicity of the original drugs and enhance their therapeutic efficacy. Pharmaceutical companies can leverage this opportunity to upgrade the technology of original high-docking-threshold compounds or shift their focus to more cutting-edge technology categories, such as crystal forms and drug combinations. Strengthening collaboration with research institutions in this field could help fill existing gaps. Importantly, the findings of this study hold significant implications for drug development, particularly in terms of potential clinical trials and market applications. The identified compounds and their unique scaffolds could serve as valuable starting points for the development of more effective and safer URAT1 inhibitors, addressing the limitations of current treatments and meeting the growing clinical demand.

## Supporting information

S1 File Search patent results. (XLSX)
